# Ellis van Creveld syndrome with unusual association of essential infantile esotropia

**DOI:** 10.4103/0974-620X.60017

**Published:** 2010

**Authors:** D. Das, G. Das, T. K. S. Mahapatra, J. Biswas

**Affiliations:** Departments of Ophthalmology and Pediatrics, R. G. Kar Medical College, Kolkata, India

**Keywords:** Postaxial polydactyly, disproportionate dwarfism, hypoplastic nails, essential infantile esotropia

## Abstract

Ellis-van Creveld syndrome is a rare short-limbed disproportionate dwarfism characterized by postaxial polydactyly, several skeletal, oral mucosal and dental anomalies, nail dysplasia and in 50-60% cases of congenital cardiac defects. It is an autosomal recessive disorder with mutations of the EVC1 and EVC2 genes located on chromosome 4p16. Patients with this syndrome usually have a high mortality in early life due to cardiorespiratory problems. We present the case of a six- month-old female infant with Ellis-van Creveld syndrome - essential infantile esotropia, which has been infrequently documented in the literature.

## Introduction

Richard W.B. Ellis of Edinburgh and Simon van Creveld of Amsterdam first described the Ellis-van Creveld syndrome (EVC) in 1940. The characteristic features of this rare autosomal recessive (AR) syndrome are short-limbed disproportionate dwarfism, postaxial polydactyly of hands, dysplastic teeth, nail changes, short ribs and in 50-60% of cases congenital heart defects.[1] The incidence of EVC in general population is very low.[2-4] The prevalence of EVC varies widely in general population 1 in 60,000 live births in USA to 1 in 150,000 live births in European countries.[5] The syndrome is most prevalent in the Amish population of USA and Arabs of Gaza strip where a prevalence of 5 in 1000 live births has been reported.[5] EVC diagnosed at birth can prevent various complications that are responsible for high mortality in early life.

## Case Report

**Figure 1:**
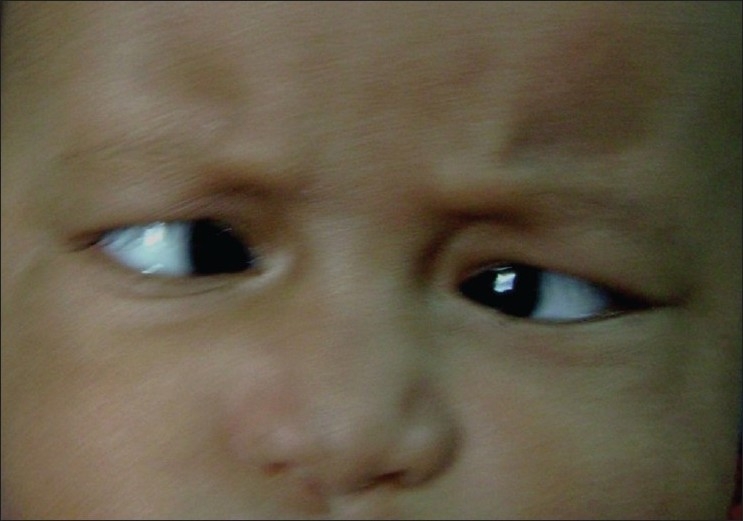
Clinical photograph showing essential infantile esotropia (OD)

**Figure 2:**
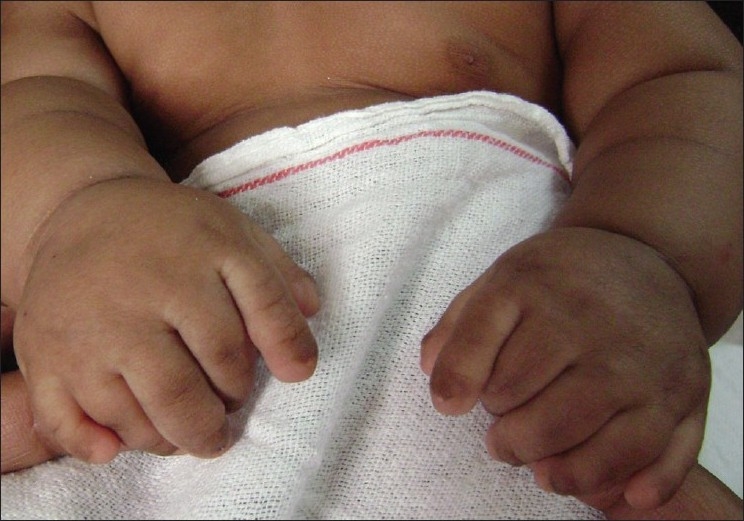
Clinical photograph showing postaxial polydactyly of both hands

A six-month-old female infant presented to us with bilateral polydactyly of hands and inward deviation of the right eye (OD). She was delivered normally at term, was the only daughter of a 28-year-old healthy father and 22-year-old healthy mother of first-degree consanguinity. No other family members had similar hand deformity or defect in the position of eyeball. Ocular examination revealed right esotropia of about 90 PD deviation with left eye fixating and cross fixation on side gaze [Figure 1]. She had no abduction deficit. Nystagmus was absent. Cycloplegic refraction in both eyes was +1.0 D. Direct Ophthalmoscopy of both eyes revealed no abnormality. On physical examination, her weight was 3.8 kg, height 48.5cm and head circumference 36 cm (all below 2SD). The chest circumference was 31 cm (below 2SD) and trunk of normal size. Each upper limb was 20 cm, while each lower limb was 16.5 cm (both below 2SD). The arm span was 43 cm (below 2SD). The arms and legs were short, clubby and shortened out of proportion to the trunk. The hands were wider than normal and feet were square shaped. Bilateral postaxial polydactyly of hands were present [Figure 2]. Her digits were proportionately short compared to her hands. Fingernails and toenails were markedly hypoplastic, thin and were set deeper. She had a fusion of the upper lip to the maxillary gingival margin that produced a V-notch in the middle of the lip and had a lip tie.

**Figure 3:**
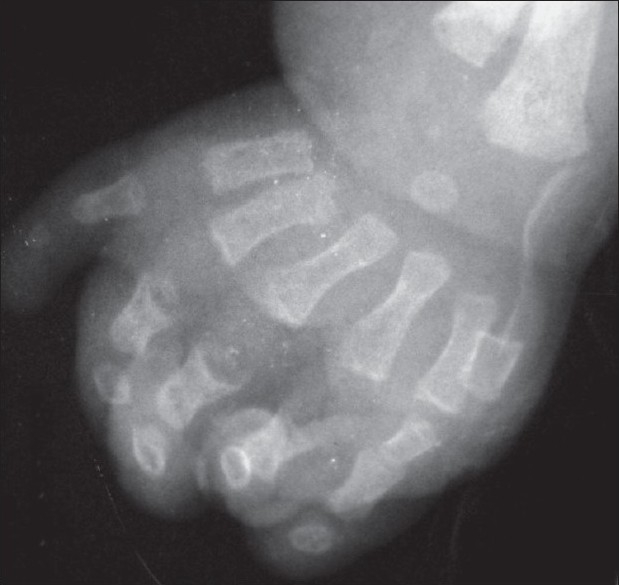
Radiograph showing shortening of distal and middle phalanges as compared to proximal ones

**Figure 4:**
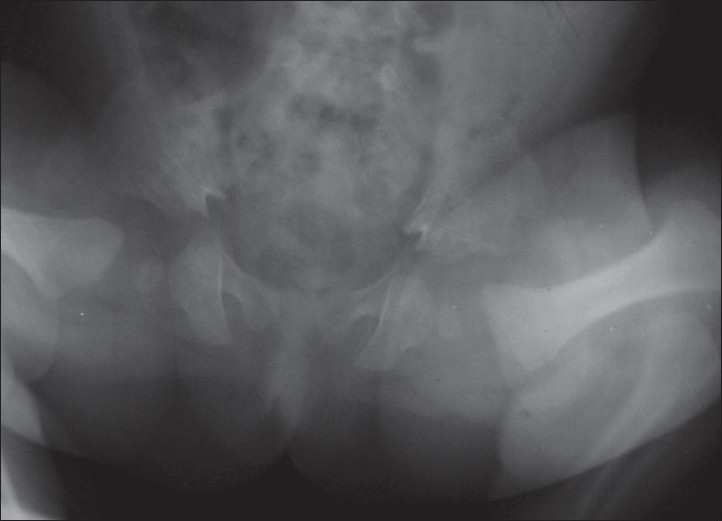
Radiograph showing bilateral short iliac wing and horizontal acetabulum with medial spurs

Oral examination revealed serrated alveolar margins without any erupted tooth. Cardiological evaluation including echo and electrocardiography revealed no abnormality. The biochemical, routine hematological and urine tests were within normal range. Radiological investigations showed postaxial polydactyly, short distal and middle phalanges compared to the proximal ones [Figure 3], short iliac wing and horizontal acetabulum with medial spurs [Figure 4]. Carpal development was delayed. Abdominal, renal and lung ultrasonogram were normal. In our case, clinical and radiological features were suggestive of EVC with essential infantile esotropia OD.

## Discussion

EVC is also called chondroectodermal dysplasia, characterized by a wide spectrum of congenital anomalies that include polydactyly, chondrodystrophy, ectodermal dysplasia, and congenital cardiac anomalies.[1] It is inherited as an autosomal recessive disorder with mutations of the EVC1 and EVC2 genes positioned in a head-to-head configuration on chromosome 4p16.[3] Recently, EVC has been included in a new class of human genetic disorders called 'ciliopathies,' where the underlying defect may be dysfunctional molecular mechanism in the primary cilia of cells.[6] Parental consanguinity has been confirmed in about 30% of cases.[4] The phenotype of this syndrome is variable and it affects multiple organs. The principal clinical features of EVC are summarized in Table 1.[1,7]

**Table 1:** Principal clinical features of EVC[1,7]

Chondrodystrophy (most common) Disproportionate dwarfism-distal limb shortening Polydactyly (constant finding) Bilateral and postaxial- hand most cases but in feet occasionally Hidrotic ectodermal dysplasia (observed in many) Nails- hypoplastic or dystrophic and friable Teeth- partial anodontia /small or conical teeth/enamel hypoplasia Hair- Sparse and fine Congenital cardiac anomalies (in 50% cases) Most common atrial septal defect leading to common atrium or Ventricular septal defect/patent ductus arteriosus/valve defects

Some inconstant clinical features of EVC are genu valgum, curvature of humerus, pectus carinatum with a long narrow chest and short poorly developed ribs, hypoplastic penis, cryptorchidism, vulvar atresia, nephrocalcinosis, renal agenesis, central nervous system anomalies, hematological abnormalities, congenital cataract, strabismus and retinitis pigmentosa.[1,8-10]

Typical radiological findings in EVC include acromesomelia (relative shortening of the distal and middle segment of the limbs), shortening of distal and middle phalanges than proximal phalanx, ulnar side polydactyly, carpal fusion, short ribs, small iliac crest and siatic notches, valgus deformity of the knee.[5]

Essential infantile esotropia is an early-acquired esotropia of unclear etiology in a neurologically normal infant. The characteristic features are onset between birth to six months of age, more than 30 PD angle of deviation, alternate or preferential fixation and nystagmus without abduction deficit.[11] After extensive MEDLINE search, we observed that the association of essential infantile esotropia with EVC has been rarely reported in the literature. EVC should be differentiated from some clinically similar conditions listed in Table 2.[7]

**Table 2:** The differential diagnosis of Ellis van Creveld syndrome [7]

Prenatally (short rib-polydactyly group) Saldino-Noonan syndrome Verma-Naumoff syndrome Beermer-Langer syndrome Postnatally Jeune dystrophy McKusick-Kaufman syndrome-hydrometrocolpos Weyers syndrome-acrodental dysostosis

In the present case bilateral postaxial polydactyly of hands, hypoplastic nails of hand and feet, short stature, alveolar ridges were suggestive of EVC. The rare features of this case were the essential infantile esotropia and horizontal acetabulum with medial spurs. Management of cases with EVC is mostly symptomatic and multidisciplinary. Some studies report growth hormone deficiency in EVC patients and highlight treatment with growth hormone for favorable result in growth of those patients.[12] Respiratory distress due to narrow chest and heart failure are causes of high mortality in early life. Early treatment can prevent various orthopedic and dental complications.

In our case, we planned to do recession of medial recti in both eyes for binocularity, but despite our best efforts the parents were not convinced about early surgical procedure in the infant.
